# Metabolonote: A Wiki-Based Database for Managing Hierarchical Metadata of Metabolome Analyses

**DOI:** 10.3389/fbioe.2015.00038

**Published:** 2015-04-07

**Authors:** Takeshi Ara, Mitsuo Enomoto, Masanori Arita, Chiaki Ikeda, Kota Kera, Manabu Yamada, Takaaki Nishioka, Tasuku Ikeda, Yoshito Nihei, Daisuke Shibata, Shigehiko Kanaya, Nozomu Sakurai

**Affiliations:** ^1^Department of Technology Development, Kazusa DNA Research Institute, Kisarazu, Japan; ^2^National Bioscience Database Center (NBDC), Japan Science and Technology Agency (JST), Tokyo, Japan; ^3^RIKEN Center for Sustainable Resource Science, Yokohama, Japan; ^4^Department of Research & Development, Kazusa DNA Research Institute, Kisarazu, Japan; ^5^Graduate School of Information Science, Nara Institute of Science and Technology, Ikoma, Japan

**Keywords:** metabolomics, metadata management system, Semantic MediaWiki, metadata sharing, data compatibility

## Abstract

Metabolomics – technology for comprehensive detection of small molecules in an organism – lags behind the other “omics” in terms of publication and dissemination of experimental data. Among the reasons for this are difficulty precisely recording information about complicated analytical experiments (metadata), existence of various databases with their own metadata descriptions, and low reusability of the published data, resulting in submitters (the researchers who generate the data) being insufficiently motivated. To tackle these issues, we developed Metabolonote, a Semantic MediaWiki-based database designed specifically for managing metabolomic metadata. We also defined a metadata and data description format, called “Togo Metabolome Data” (TogoMD), with an ID system that is required for unique access to each level of the tree-structured metadata such as study purpose, sample, analytical method, and data analysis. Separation of the management of metadata from that of data and permission to attach related information to the metadata provide advantages for submitters, readers, and database developers. The metadata are enriched with information such as links to comparable data, thereby functioning as a hub of related data resources. They also enhance not only readers’ understanding and use of data but also submitters’ motivation to publish the data. The metadata are computationally shared among other systems via APIs, which facilitate the construction of novel databases by database developers. A permission system that allows publication of immature metadata and feedback from readers also helps submitters to improve their metadata. Hence, this aspect of Metabolonote, as a metadata preparation tool, is complementary to high-quality and persistent data repositories such as MetaboLights. A total of 808 metadata for analyzed data obtained from 35 biological species are published currently. Metabolonote and related tools are available free of cost at http://metabolonote.kazusa.or.jp/.

## Introduction

Vast amounts of metabolome data are produced daily by high-throughput mass spectrometers; however, publication of these data and their reuse are limited. Metabolomics is a technological area that focuses on comprehensive detection of small molecules derived from the biosynthesis of living organisms (Oliver et al., 1998; Dunn, 2008). It is applied to a wide range of fields such as biology (Kopka et al., 2004; Khoo and Al-Rubeai, 2007; Mashego et al., 2007), exploration of biomarkers in medical sciences (Jansson et al., 2009; Koulman et al., 2009), quality evaluation of foods (Pongsuwan et al., 2008; Fitzgerald et al., 2009), and assessment of environmental pollution (Lin et al., 2006; Krauss et al., 2010). Mass spectrometry (MS) is one of the popular choices for compound detection because of its advantage in factors such as sensitivity and throughput (Zhang et al., 2012; Dunn and Hankemeier, 2013). However, in terms of publication of the experimental data in public databases, metabolomics lags far behind the other “omics” such as transcriptome and proteome (Griffin and Steinbeck, 2010). Several metabolomics-specific reasons appear to be associated with this issue. First, a vast amount of metadata – detailed information about data – usually accompanies the metabolome data, which are obtained from complicated procedures and conditions of metabolite extraction, instrumental analysis, and computational processing. Therefore, standards for minimal information about metadata description are still under discussion (Fernie et al., 2011; Griffin et al., 2011). One major data generation bottleneck is identification of metabolites (Wishart, 2009; Bowen and Northen, 2010), which causes finalization of the data analysis to take an extended amount of time. During this period, a part of the complicated metadata may be lost or become difficult to trace. Second, many databases specially constructed for each purpose already exist, and the required metadata are different for each. Consequently, submitters have to describe complicated metadata according to each metadata format, even when the data are derived from the same raw data. Finally, the most serious issue is that reuse of published metabolome data is quite limited because determination of the comparability of data for further analysis is difficult for database readers, owing to the complexity of the experimental procedures. These issues make general researchers less motivated to publish their own data in the databases.

The issues outlined above have to be resolved in order to improve publication and dissemination of metabolomic data. The standardization of minimum information for metadata description has been under discussion since 2005 by the Metabolomics Standards Initiative (Fiehn et al., 2007), and continued by COSMOS since 2012 (Steinbeck et al., 2012; Salek et al., 2013a); thus, standardization is likely to be resolved in the near future. Recent progress in metabolite annotation tools (Horai et al., 2010; Fukushima and Kusano, 2013; Kind et al., 2013; Hufsky et al., 2014) will accelerate the production of data. Use of metadata management tools such as ISAcreator (Rocca-Serra et al., 2010) and XperimentR (Tomlinson et al., 2013) will reduce the risk of metadata loss. On the other hand, sharing of metadata is still problematic. ISA-Tab, which is designed for metadata sharing (Sansone et al., 2008; Rocca-Serra et al., 2010), is utilized in the metabolome data repository MetaboLights (Steinbeck et al., 2012; Haug et al., 2013). However, MetaboLights does not provide APIs for semantic search of the metadata. To reuse metadata from other systems, developers have to reconstruct the metadata from ISA-Tab files, which are retrieved in a synchronized manner from the MetaboLights website. MetabolomeXchange^1^, a portal website that gathers metabolome data resources published in databases such as MetaboLights, GMD (Kopka et al., 2005), and Metabolomics Workbench^2^, manages only limited items of metadata, with details remaining in the original databases. Therefore, cross-database searching for metadata details is not provided in the set of APIs they provide. The issue of metadata sharing is one of the reasons why determination of comparable data is still difficult for database readers. Consequently, no increase in the motivation of researchers to publicize data can be expected.

To overcome these fundamental issues in metabolomics, we developed a Semantic MediaWiki-based database called Metabolonote. We briefly mentioned Metabolonote in a previous article about our web portal KOMICS, in which we introduced the metabolomics tools and databases that we have developed (Sakurai et al., 2014). In this paper, we report on it in more detail. The ideas underlying the development of Metabolonote include complete separation of the management of metadata from that of data, and permission to attach related information to the metadata. Metabolonote is a metadata-specific database; thus, submitters (the majority of whom are assumed to be general researchers who generate the data and want to deposit and publicize them) can start describing complicated metadata without the substance of the experimental data. Further, an easy-to-use wiki system helps submitters to reduce loss of metadata. The metadata-specific database is implemented with APIs for semantic searching and retrieving of metadata, and enables computational sharing of metadata among multiple databases without invading their own property values. Delegation of metadata management to Metabolonote facilitates the construction of novel databases by database developers. The permission to attach related information to the metadata enables submitters to publish their knowledge of other data that are comparable to their data. We believe that this will promote the reuse of metabolome data and motivate researchers to publish their data. Thus, these features are advantageous for all submitters, readers, and database developers.

## Materials and Methods

**Figure 1:**
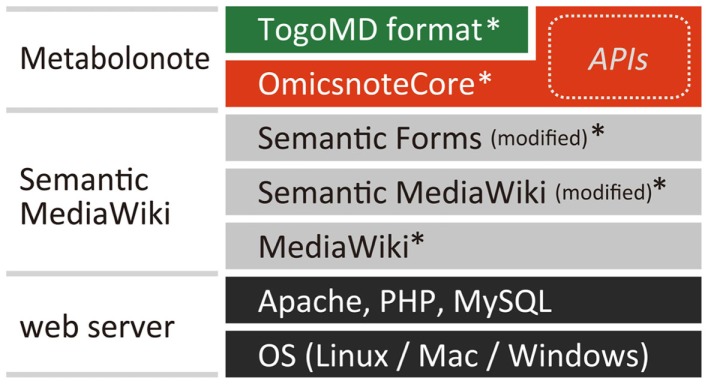
**System architecture of Metabolonote**. The core system of Metabolonote, OmicsnoteCore is developed as an extension of Semantic MediaWiki, an extension of the content management system MediaWiki, which is written in PHP. A part of SemanticMediaWiki and its extension Semantic Forms was modified. By defining other corresponding formats apart from TogoMD, the system can manage other metadata. OmicsnoteCore implements TogoMD format-dependent and -independent APIs for semantic search and retrieval of metadata from other systems. The program and settings files for implementing Metabolonote in a local server (*) are available from the Metabolonote website.

Metabolonote is constructed as an extension called “OmicsnoteCore” for Semantic MediaWiki^3^, a content management system written in PHP, and a set of wiki page data (Properties, Templates, and Forms) prepared based on the “Togo Metabolome Data” (TogoMD) metadata format (described in the Section “The TogoMD Format”) (Figure 1). Functions related to form-editing, property displaying, and data storage in the extensions of MediaWiki^4^, Semantic MediaWiki, and Semantic Forms, are partially modified. The Metabolonote website is currently running on Red Hat Enterprise Linux Server release 5.6 with Apache 2.2, PHP 5.3, and MySQL 5.0. A sample program called “MNSearchDemo,” which includes practical examples of API usage for semantic searching and retrieving of metadata, is written in PHP. These programs and settings files are available free of cost at Metabolonote’s help page^5^.

## Results

### System architecture

We chose Semantic MediaWiki as the base system for Metabolonote because the following features satisfy our metadata management requirements: (1) The contents can be edited via a web browser. (2) The contents can be shared as webpages. (3) APIs for computationally searching and retrieving the contents can be implemented. (4) Users’ inputs are managed as values of properties in a wiki page; this data structure is suitable for managing a set of metadata consisting of several defined items that need to be described. (5) The form-editing interface provided by the extension Semantic Forms is suitable for inputting the formatted metadata. (6) The concept of subpages in MediaWiki, in which a hierarchy of wiki pages is represented as a directory path by a URL separated by a slash “/,” is suitable for representing the hierarchy of metadata (see The TogoMD Format) and to provide a way to uniquely access each metadata. Finally (7), additional information can be attached to the metadata in HTML format or MediaWiki’s proprietary markup language. Therefore, we constructed the core system of Metabolonote, OmicsnoteCore, as an extension of Semantic MediaWiki (Figure 1). OmicsnoteCore provides functions for managing the hierarchical structure of metadata, user access controls, and APIs to search and retrieve the metadata. We defined a format “TogoMD” (see below) as a set of metadata for use on the OmicsnoteCore. However, OmicsnoteCore can be used to manage other metadata apart from TogoMD simply by submitting the definition of that set of metadata.

### The TogoMD format

**Figure 2:**
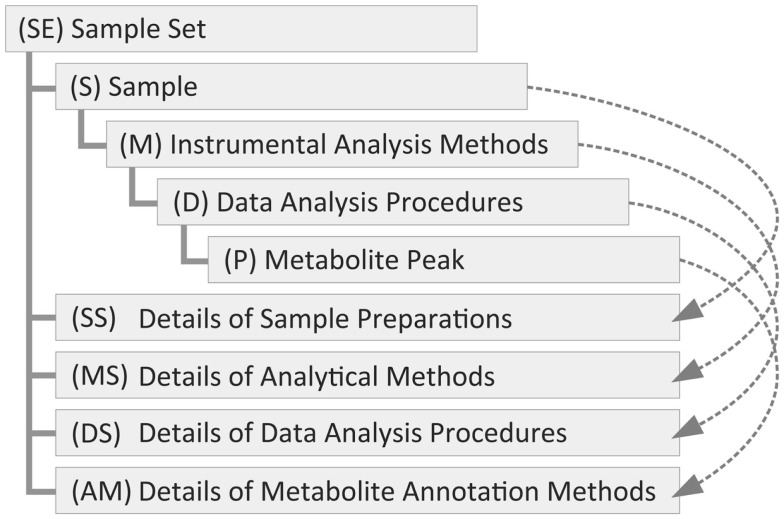
**Hierarchy of the metadata classes of the TogoMD format**. The metadata of metabolome analysis is divided into various classes; specifically, a class for the purpose of study information with a set of samples (SE), samples (S), analytical methods (M), and data analysis (D). These classes constitute a tree structure. We define classes for commonly used (shared) procedures for sample preparation details (SS), analytical method details (MS), data analysis details (DS), and annotation method details (AM) under the top-level class. Their instances are referred from the instances of the other classes (dashed arrows). The data class for peak information (P) is not used in Metabolonote. The parentheses are the prefixes of their instance ID.

To manage the metadata in Metabolonote, we defined a novel metadata and data format called the TogoMD format. This novel format was conceptualized with the following considerations. The metabolomic metadata can be separated into hierarchical classes, specifically, a class for information about the purpose of the study, a class for sample preparation information, a class for analytical method information, and a class for data analysis information (Figure 2). In general, multiple samples are used per study; for example, biological replications of treatment and control groups. A sample can be analyzed in several ways and multiple times; for example, by liquid chromatography–MS, gas chromatography–MS, NMR, and their analytical replications. The raw data generated by each analysis can be analyzed in several ways. For instance, computational tools and procedures should be different when the data are used as fingerprints, for metabolite annotations to make metabolic profiling data, or used for getting tandem mass spectrum data. Each set of data generated through the process should be related to each class at a different level of the hierarchy. For instance, the raw data generated by the analytical apparatus should be related to the analytical method class, and the processed data should be related to the data analysis class. To share the metadata among outside systems, a method for uniquely accessing the metadata at each level of the hierarchy, possibly by unique identifiers (IDs), is required. We first evaluated the utility of ISA-Tab, which is used in MetaboLights (Sansone et al., 2008; Rocca-Serra et al., 2010). In ISA-Tab, the hierarchy of the classes mentioned above is connected with sample names and names assigned to the protocols (a set of common procedures). Therefore, control of the nomenclature of these names is required to establish a method to uniquely and computationally access the metadata at each level of the hierarchy, and modifications of the predefined format such as addition of an extra column for IDs is possibly required to control it well. Therefore, we defined TogoMD, which includes a rule for ID assignment (details are given in Systematic ID Design for the Metadata). Similar to the ISA-Tab protocol, frequently utilized procedures can be written as referent information. As each metadata has description and comment fields for free description by the submitter, almost the same metadata as those in ISA-Tab can be described in accordance with the recommendation of MSI (Fiehn et al., 2007) and also should be conducted in line with that of COSMOS (Steinbeck et al., 2012) Working Package 2 in the future. The relationships between the description fields of ISA-Tab and TogoMD used in Metabolonote are shown in Table S1 in Supplementary Material. Most of the fields in TogoMD, including author description, are simpler than those of ISA-Tab. TogoMD also defines formats for the data files. The details of the formats are described in the online help at the Metabolonote website^6^.

### Systematic ID design for the metadata

To provide a simple way to uniquely access the metadata at each level of the hierarchy, an ID assignment rule for the metadata is a prerequisite in OmicsnoteCore. A metadata ID consists of alphabetic prefixes representing the class of the metadata, followed by a digit part representing the instance of the class, then an underscore connecting the instances. In the case of TogoMD, the following four classes and the prefixes shown in the parentheses are defined. The class for study purpose consists of a set of samples (SE) that corresponds to Study in ISA-Tab, with sample information (S), analytical methods (M), and data analysis (D) (Figure 2). A data class for the detected peaks (P) is also defined subsequent to class D; however, it is not used in Metabolonote. As an instance, a metadata ID related to an analytical method is represented as SE1_S01_M01, and that for the processed data derived from the analysis data is represented as SE1_S01_M01_D01. The instances of the classes for the metadata of frequently referred and shared procedures, specifically, detailed information about sample preparation (SS), analytical methods (MS), data analysis (DS), and metabolite annotation methods (AM), are placed beneath the instance of SE; for example, SE1_MS1 (Figure 2, dashed arrows). The metadata are displayed in web browsers by accessing the URL with the IDs. In this case, the separators of the hierarchy level are replaced by a colon plus slash “:/” for the top-level class and a slash “/” for the rest. For example, http://metabolonote.kazusa.or.jp/SE1:/S01/M01 for SE1_S01_M01. The digit part of the top-level instance (SE) is managed and assigned by the administrator of Metabolonote, whereas those of the other instances are assigned by the submitter themselves. Any array of digits can be assigned unless the same prefix plus array of digits are placed under the same metadata ID. The set of ID rules allows the submitters to assign meaningful digits to their own data. For example, they can assign S01–S03 for triplicates of drug-treated samples, S11–S13 for the controls, and S91–S95 for authentic compounds. The only thing the administrator of the system has to do with regards to ID management is to control the uniqueness of the ID of the top-level instance. The concatenation of the metadata with the separators facilitates clear understanding of the hierarchy of the metadata. More detailed information about ID nomenclature is given on the Metabolonote help page^7^.

### Editing and publication of metadata

**Figure 3:**
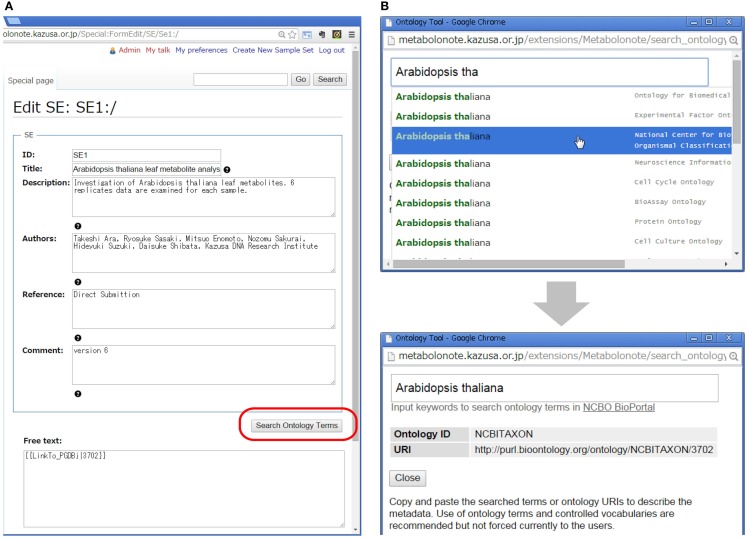
**Form-editing window of Metabolonote**. **(A)** Metadata and attachment of additional information can be edited using forms. **(B)** The function “Search Ontology Terms,” available at the button represented by the red round circle in **(A)**, is used to search for ontology terms in BioPortal in NCBO.

Creation and editing of the metadata can be done via web browsers without installing any special software. Figure 3A shows a form-editing interface for an SE-instance with a minimized number of description items in TogoMD format. The submitters can search ontology terms in the BioPortal at the National Center for Biomedical Ontology (NCBO) (Whetzel et al., 2011) by clicking the “Search Ontology Terms” button (Figure 3B). Submitters currently do not have to use the proper ontology terms for fields such as species names, but the function helps to reduce incidents of misspelling of specific terms, and helps to query the ID in the Taxonomy database of the National Center for Biotechnology Information (NCBI) (Federhen, 2012). In the “Free text” area, submitters can attach additional information in HTML or MediaWiki’s markup language. This area can be used, for example, to make links to the record of other databases in which data related to the metadata are deposited. Metabolonote provides several wiki templates to facilitate the creation of links by users. To prevent inappropriate writing and the creation of links to malicious websites, only system administrators are currently allowed to change the public/private state of the metadata.

### Exemplary usage as a hub of data resources

**Figure 4:**
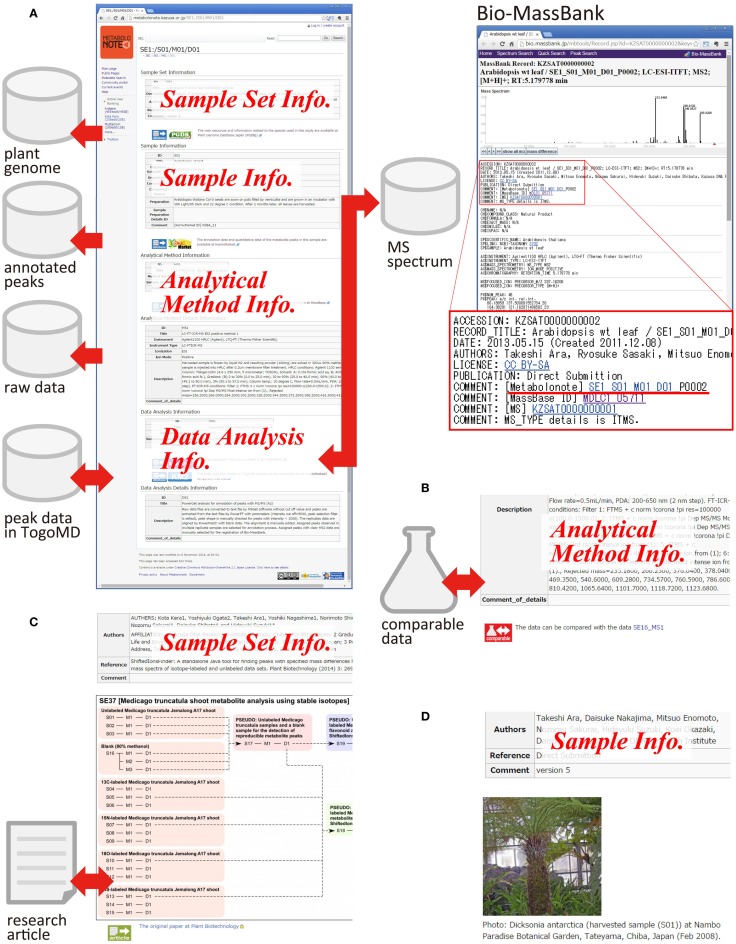
**Metadata as a hub for other data resources**. Examples of the metadata are shown: **(A)** Metadata for processed data obtained from *Arabidopsis* (SE1_S01_M01_D01, the original metadata are available at http://metabolonote.kazusa.or.jp/SE1:/S01/M01/D01). A reciprocal link is made with the mass spectrum database Bio-MassBank. **(B)** Metadata with a link to the comparable data (SE12_MS1, the original metadata are available at http://metabolonote.kazusa.or.jp/SE12:/MS1). **(C)** Metadata with images representing analytical procedures (SE37, the original metadata are available at http://metabolonote.kazusa.or.jp/SE37:/). The metadata are used as Supplementary Material in the research article. **(D)** Metadata with a picture of the sample (SE4, the original metadata are available at http://metabolonote.kazusa.or.jp/SE4:/).

By adding extra information in the free text area, a metadata can become a hub with rich information about related data resources. Figure 4A is an example of metadata of computationally analyzed data obtained from the liquid chromatography–MS analysis of leaves of a model plant, *Arabidopsis* (SE1_S01_M01_D01). Several links to the other databases are attached. In Metabolonote, all of the metadata at the upper levels of the hierarchy are displayed as a single webpage. A link to PGDBj (Asamizu et al., 2014), which provides integrated information around plant genome data, is attached to the metadata of the sample set (SE). At the metadata of the samples, a link to the record of KomicMarket (Sakurai et al., 2014), the peak annotation database is present. For the analytical methods metadata, a link to the corresponding raw data in MassBase (Sakurai et al., 2014) is present. Links to two databases are attached to the data analysis procedures metadata; namely, Bio-MassBank^8^, the mass spectrum library from organisms, and KomicMarket2’s temporary website (KM2)^9^, which provides peak annotation data in TogoMD format. The metadata in Metabolonote is referred from the record pages in Bio-MassBank by a URL link, which complements the detailed description of metadata in Bio-MassBank. Figure 4B shows an attachment of a link to the other data resource that is comparable. Figure 4C is an example of the attachment of an image representing the process of data generation. Detailed information about the process in Metabolonote is referred as a Supplementary Material from Kera et al. (2014). A link to the article website is also attached. Figure 4D is another example of attachment of pictures for aiding understanding about the analyzed samples. This information aids in the reuse of published data by the readers. As shown in Figure 4, the metadata prepared by the submitters becomes a hub of related data resources, and give rich information to the readers.

### APIs for searching and retrieving the metadata

Metabolonote provides APIs for searching semantically and retrieving the metadata, by which computational sharing of metadata from the other systems is realized. The description of items in a metadata class is designated as “property” in Semantic MediaWiki. The values of the property can be searched by the APIs. Two APIs for semantic search (search for properties that have specific values) and 12 for data retrieval are currently provided. The “Metadata Search” function in Metabolonote was developed by internally using these APIs. The KM2 website, which distributes the peak annotation data in TogoMD format, was also developed using the APIs. No metadata management system is implemented in KM2, and KM2 only manages the data with their corresponding metadata IDs. When the metadata are searched for by the semantic search APIs, a list of metadata IDs is returned. Then, KM2 displays the results for those data whose IDs are on the list. Sharing of metadata via APIs significantly aids the database developers by releasing them from designing and developing a metadata management system for their own web applications. A sample PHP program called “MNSearchDemo” is available at the Metabolonote website. MNSearchDemo is a practical example of API usage, and by which developers can create a simple database like KM2 in local servers.

### Current statistics of metadata

The metadata for 808 sets of analysis data (D) obtained from 420 experimental analyses (M) of 149 samples (S) examined in 51 sample sets (SE) consist of 35 biological species, including plants, algae, bacteria, and animals, are currently published in Metabolonote. Six analytical methods (MS) have links to the comparable data. Links to the eight other databases, namely, MassBase, KomicMarket, Bio-MassBank, PGDBj, KM2, PRIMe (Sakurai et al., 2013), MetaboLights, and MetabolomeExpress (Carroll et al., 2010), are included.

## Discussion

### Benefits of metadata-centric resource management

By separating the management of metadata from that of data, and permitting the attachment of related information to the metadata, Metabolonote provides benefits to all submitters (who are assumed to be general researchers who generate and deposit their data), readers, and database developers. The submitters become motivated to publish their data by attaching additional information that makes their work attractive. Piwowar et al. (2007) surmised that increases in the citation of research articles for which the data are published can be attributed to increase exposure of the work. However, their statement is based on microarray data, which is more easily comparable than metabolome data. Even when standardized descriptions are obligated by the journals (Salek et al., 2013b), issues of data comparability remain because of the complexity of experimental conditions. It is not practicable to annotate all comparability between data stored in data repositories. Thus, provision of comparability information by the submitters themselves is one practical solution to this problem that can lead to increased use of their data and citation of their work. When rich information is provided by submitters, it is advantageous for the readers to understand about the data and to find related data resources, especially comparable data, by which reuse of metabolome data will be promoted. For the database developers, delegation of the metadata management system to Metabolonote is advantageous as it aids in the development of novel databases like KM2. If the metadata sharing became common, submitters would be released from repeated description and management of their metadata in multiple databases. Metadata that are described in a uniform format helps readers to understand the data. Separate management of metadata and experimental raw data, and then accessibility to them, may also help an increasing requirement for data sharing in the biomedical research field, where unwillingness to share data is of major issues (Piwowar et al., 2008; Wang et al., 2014).

### Complementary usage with the other repositories

The aspect of Metabolonote as a support system for completion of metadata is complementary to other curated, high-quality, and persistent repositories such as MetaboLights. Metabolonote allows submitters to publish immature descriptions of metadata. This promotes publication of metabolome data that were not previously considered publishable owing to inadequateness of the metadata. Depending on the purpose of a study, the required metadata for the published data should be different. For example, in the case of analysis of species-specific MS/MS spectrum, information about species and the type of MS would be sufficient if the data are abundantly provided. Conversely, in the case of comparison of metabolic profiles, further detailed metadata such as extraction and chromatography separation are essential. Permission to publish immature metadata is advantageous for submitters as it allows them to get feedback or requests from readers for more detailed metadata. Providing all of their data with completed metadata is a burden for submitters. However, when they receive requests from readers who are interested in their work, they can focus on curation and enrichment of the metadata for the requested data. Such requests from readers should help to significantly motivate the submitters. Further, if metadata improved through these processes become a sufficiently high-quality, then the submitters can deposit them to MetaboLights. They can make a link to MetaboLights from the metadata in Metabolonote, and readers can then judge for themselves the quality of the metadata.

### Metadata-specific system

To the best of our knowledge, Metabolonote is the first database specified for managing metabolomics metadata. Most life science databases manage the metadata along with the data. There are several metadata-specific systems, such as NCBI BioProject (Barrett et al., 2012) and GEOmetadb (Zhu et al., 2008). These are aimed at managing vast amounts of data that have been deposited. BioSamples Database (Gostev et al., 2012) is a metadata-specified system for biological samples and was developed to connect multiple-omics data in the European Bioinformatics Centre. The concept of Metabolonote in metadata-centric management of various data is similar to that of BioSamples. However, BioSamples does not support description and sharing of the metadata of metabolome experiments, and is not targeted at authentic compounds that can be analyzed in metabolite identification. Other metadata-centric systems can be found in the field of digital library management. Free software such as DSPACE^10^ and Islandora^11^ are well known, but our investigations reveal that there is no system that supports hierarchical structured metadata. A portal for metabolomics databases, MetabolomeXchange is a metadata-specific system because it does not manage experimental data. However, as mentioned in the Introduction, cross-database searching by detailed metadata is not yet supported. Therefore, from all appearances, Metabolonote is a novel database that manages rich metadata separately from the management of experimental data, and is aimed at the sharing of hierarchical structured metadata. As any kind of data can be related to the metadata, OmicsnoteCore may be highly suited to manage metadata from multiple-omics analyses and study fields where novel analytical methods are frequently applied. OmicsnoteCore is also used as a laboratory data management system in some laboratories by defining other metadata formats apart from TogoMD.

### Future perspectives

To assist knowledge discoveries through intensive analysis of vast amounts of metabolome data, further improvements are required. For advanced use of metabolome data, detailed facet search with various parameters such as parts of the body, sampling environment, type of chromatography column, and name of data analysis tool, will be essential in the future. However, to realize this we have to tackle a contradictory matter, namely, subdivision of metadata description items and their efficient collection. We are currently in discussions about a format that will extend TogoMD to describe sub-items in the fields for “description” and “comment.” The description of sub-items should not be mandatory but should be easy to fill in by the submitters or by the article curators. An easy-to-use and intuitive user interface should also be developed. Another requirement in order to realize facet searching is preparation of semantic web technologies. Semantic search APIs are already implemented in Metabolonote. However, for advanced search across resources on other databases, the metadata should be queried by a semantic query language such as SPARQL^12^. For this purpose, preparation of the metadata in Resource Description Framework (RDF)^13^ and preparation of a set of necessary ontologies and controlled vocabularies by using pre-existing ones such as Mass Spectrum ontology^14^ are currently underway. As mentioned in the Section “Complementary Usage with the Other Repositories,” requests and feedback from readers help submitters to improve the quality of the metadata. A system for efficient communication between submitters and readers is therefore being considered.

## Conflict of Interest Statement

The authors declare that the research was conducted in the absence of any commercial or financial relationships that could be construed as a potential conflict of interest.

## Supplementary Material

The Supplementary Material for this article can be found online at http://journal.frontiersin.org/article/10.3389/fbioe.2015.00038

## Footnotes

^1^http://metabolomexchange.org/

^2^http://www.metabolomicsworkbench.org/

^3^http://semantic-mediawiki.org/

^4^http://www.mediawiki.org/

^5^http://metabolonote.kazusa.or.jp/Help:Contents

^6^http://metabolonote.kazusa.or.jp/TogoMetabolomeDataFormat

^7^http://metabolonote.kazusa.or.jp/Help:Data_Structure_ID

^8^http://bio.massbank.jp/

^9^http://nedo3.kazusa.or.jp/km2_tmp/

^10^http://www.dspace.org/

^11^http://islandora.ca/

^12^http://www.w3.org/TR/sparql11-overview/

^13^http://www.w3.org/TR/rdf11-concepts/

^14^http://bioportal.bioontology.org/ontologies/MS
