# Supplementary material for: Metabolonote: A Wiki-Based Database for Managing Hierarchical Metadata of Metabolome Analyses
Source: Front Bioeng Biotechnol. 2015 Apr 7;3:38. doi: 10.3389/fbioe.2015.00038 (PMC4388006; doi:10.3389/fbioe.2015.00038)
Supplement: Supplementary file 1 [file Table_1.PDF]

Supplementary Material

Metabolonote: A wiki-based database for managing hierarchical metadata of metabolome analyses

Takeshi Ara[1,2,†], Mitsuo Enomoto[1,2], Masanori Arita [2,3], Chiaki Ikeda[1,2], Kota Kera[4], Manabu Yamada[1,2], Takaaki Nishioka[2,5], Tasuku Ikeda[2,5], Yoshito Nihei[2,5], Daisuke Shibata[1], Shigehiko Kanaya[2,5], Nozomu Sakurai[1,2,*]

[1] Department of Technology Development, Kazusa DNA Research Institute, Kisarazu, Japan
[2] JST, National Bioscience Database Center (NBDC), Tokyo, Japan
[3] RIKEN Center for Sustainable Resource Science, Yokohama, Japan
[4] Department of Research & Development, Kazusa DNA Research Institute, Kisarazu, Japan
[5] Nara Institute of Science and Technology, Graduate School of Information Science, Ikoma, Japan

[*] **Correspondence:** Nozomu Sakurai, Metabolomics Team, Department of Technology Development, Kazusa DNA Research Institute, 2-6-7 Kazusa-kamatari, Kisarazu, Chiba 292-0818, Japan.
sakurai@kazusa.or.jp

[†] **Present address:** KAGOME Tomato Discoveries Lab., Graduate School of Agriculture, Kyoto University, Kyoto, Japan

1. Supplementary Tables

1.1. Supplementary Tables

Supplementary Table 1. Relationships between the description fields in TogoMD used in Metabolonote and those in ISA-Tab for MetaboLights.

**TogoMD fields used in Metabolonote**		**Relationship[a]**	**Entities in ISA-Tab for MetaboLights**
SE	**Sample Set Information**	~	**Investigation and Study[b]**
	ID	~	Investigation Identifier and Study Identifier
	Title	~	Investigation Title and Study Title
	Description	=	Investigation Description and Study Description
	Authors	~	Investigation Person Last Name, Investigation Person First Name, Investigation Person Mid Initials, Investigation Person Email, Investigation Person Phone, Investigation Person Fax, Investigation Person Address, Investigation Person Affiliation, Investigation Person Roles, Investigation Person Roles Term Accession Number, Investigation Person Roles Term Source REF, Study Person Last Name, Study Person First Name, Study Person Mid Initials, Study

			Person Email, Study Person Phone, Study Person Fax, Study Person Address, Study Person Affiliation, Study Person Roles, Study Person Roles Term Accession Number, and Study Person Roles Term Source REF
	Reference	~	Study PubMed ID, Study Publication DOI, Study Publication Author List, Study Publication Title, Study Publication Status, Study Publication Status Term Accession Number, and Study Publication Status Term Source REF
	Comment	Absent	
S	**Sample Information**	~	**Study**[b]
	ID	~	Sample Name[b]
	Title	~	Sample Name[b] and Study Factor Name
	Organism - Scientific Name	=	Characteristics[Organism] of the Study "Sample Name"
	Organism - ID	Absent	
	Compound - ID	Absent	
	Compound - Source	Absent	
	Preparation (deprecated)	=	Study Protocol Description of the Protocol Name "Sample collection"
	Sample Preparation Details ID	Absent	
	Comment	Absent	
SS	**Sample Preparation Details Information**	~	**STUDY PROTOCOLS**[b]
	ID	~	"Sample collection"[c]
	Title	~	"Sample collection"[c]
	Description	=	Study Protocol Description of the Protocol Name "Sample collection"
	Comment_of_details	Absent	
M	**Analytical Method Information**	~	**Assay**[b]
	ID	~	Assay Name[b]
	Title	~	Assay Name[b]
	Method Details ID	Absent	
	Sample Amount	Absent	
	Comment	Absent	
MS	**Analytical Method Details Information**	~	**STUDY PROTOCOLS**[b]
	ID	~	"Mass spectrometry"[c]
	Title	~	"Mass spectrometry"[c]

	Instrument	~	Parameter Value[Instrument] of the Protocol Name "Mass spectrometry", and Parameter Value[Chromatography Instrument] of the Protocol Name "Chromatography"
	Instrument Type	~	Parameter Value[MS analyzer] of the Protocol Name "Mass spectrometry"[d]
	Ionization	=	Parameter Value[Ion source] of the Protocol Name "Mass spectrometry"
	Ion Mode	=	Parameter Value[Scan polarity] of the Protocol Name "Mass spectrometry"
	Description	~	Study Protocol Description of the Protocol Name "Extraction", "Chromatography", and "Mass spectrometry"
	Comment_of_details	Absent	
D	**Data Analysis Information**	~	**Assay**[b]
	ID	~	Assay Name[b]
	Title	~	Assay Name[b]
	Data Analysis Details ID	Absent	
	Recommended decimal places of m/z	Absent	
	Comment	Absent	
DS	**Data Analysis Details Information**	~	**STUDY PROTOCOLS**[b]
	ID	~	"Data transformation"[c]
	Title	~	"Data transformation"[c]
	Description	=	Study Protocol Description of the Protocol Name "Data transformation"
	Comment_of_details	Absent	
AM	**Annotation Method Details Information**	~	**STUDY PROTOCOLS**[b]
	ID	~	"Metabolite identification"[c]
	Title	~	"Metabolite identification"[c]
	Description	=	Study Protocol Description of the Protocol Name "Metabolite identification"
	Comment_of_details	Absent	

[a] The equal sign "=" signifies that the values of the fields in ISA-Tab for MetaboLights and Metabolonote can be exchanged computationally. Tilde "~" signifies that the field in Metabolonote corresponds to that in ISA-Tab for MetaboLights but the values cannot be exchanged computationally. "Absent" signifies that there is no corresponding entity in ISA-Tab for MetaboLights. [b] The entity name of ISA-Tab for MetaboLights is related to multiple fields of

TogoMD. [c] Names of the Study Protocols predefined in ISA-Tab for MetaboLights. [d] Different ontologies are used.
